# Circulating Cell-Free DNA in Hepatocellular Carcinoma: Current Insights and Outlook

**DOI:** 10.3389/fmed.2018.00078

**Published:** 2018-03-26

**Authors:** Charlotte K. Y. Ng, Giovan Giuseppe Di Costanzo, Luigi M. Terracciano, Salvatore Piscuoglio

**Affiliations:** ^1^Institute of Pathology, University Hospital Basel, Basel, Switzerland; ^2^Department of Biomedicine, Hepatology Laboratory, University of Basel, Basel, Switzerland; ^3^Department of Transplantation – Liver Unit, Cardarelli Hospital, Naples, Italy

**Keywords:** cell-free DNA, circulating tumor DNA, hepatocellular carcinoma, liquid biopsy, somatic mutations, copy number alterations, methylation

## Abstract

Over the past decade, the advancements in massively parallel sequencing have provided a new paradigm in biomedical research to uncover the genetic basis of human diseases. Integration of ‘omics information has begun transforming clinical management of cancer patients in terms of diagnostics and treatment options, giving rise to the era of precision medicine. Currently, nucleic acids for molecular profiling for patients diagnosed with hepatocellular carcinoma (HCC) are typically obtained from resected tumor materials or transplanted neoplastic liver and occasionally from biopsies. Given the intrinsic risks associated with such invasive procedures, circulating cell-free DNA (cfDNA) has been proposed as an alternative source for tumor DNA. Circulating cfDNA is a type of cell-free nucleic acid that derives from apoptotic, necrotic, as well as living eukaryotic cells. Importantly, the detection of abnormal forms of circulating cfDNA that originate from cancer cells provides a new tool for cancer detection, disease monitoring, and molecular profiling. Currently, cfDNA is beginning to be adopted into clinical practice as a non-invasive tool to monitor disease by tracking the evolution of disease-specific genetic alterations in several major cancer types. Moreover, cfDNA is demonstrating potential clinical value as a surrogate to assess the molecular makeup of tumors and to overcome the sampling biases inherent to intra-tumor genetic heterogeneity, especially in the metastatic setting. With the improvements in ‘omics and molecular biology techniques, coupled with the increasing understanding in the molecular pathogenesis of cancer, it can be anticipated that the detection and analysis of cfDNA will become more specific and sensitive and thus enable cfDNA analysis to be used as a diagnostic aid in patients with early-stage disease and perhaps even in a screening setting. In this review, we provide an overview of the latest findings on the role and potential utility of cfDNA analysis in the diagnosis, management, and screening of HCC.

## Review Criteria

Review of the literature was performed in English using the PubMed database. Search terms used included “hepatocellular carcinoma,” “liver cancer,” “circulating cell free DNA,” “circulating tumor DNA,” “plasma,” “serum,” and “liquid biopsy.”

## Introduction

Over the past decade, the advancements in next-generation sequencing (NGS) and high-throughput omics profiling have provided a new paradigm in biomedical research to uncover the epi/genetic basis of human diseases. Integration of omics information has begun to transform clinical management of cancer patients in terms of diagnostics, prevention, and treatment options, giving rise to the era of precision medicine. Compared to cancer types such as those of the breasts and the lungs, precision medicine for hepatocellular carcinoma (HCC) patients is lagging behind, owing to a number of HCC-specific challenges in clinical practice and the lack of treatment options.

Hepatocellular carcinoma accounts for >90% of liver cancers and was responsible for an estimated 782,000 new cancer cases and nearly 746,000 deaths in 2012 (1). While HCC is the sixth most common cause of cancer worldwide, it is the third most common cause of cancer deaths and has one of the highest mortality-to-incidence ratios (2). HCC usually occurs in cirrhotic livers, and the epidemiology of HCC shows marked variations between geographical regions and racial groups (3). In sub-Saharan Africa and south-east Asia, hepatitis B virus (HBV) chronic infection is endemic and accounts for the majority of HCCs diagnosed (2). In Western populations, the rising incidence of HCC is in part driven by the increasing prevalence of chronic liver diseases associated with hepatitis C virus (HCV) infection, alcohol consumption, diabetes, obesity, and non-alcoholic fatty liver disease (2, 4, 5). Other known risk factors of HCC include hereditary diseases such as hemochromatosis and α-1-antitrypsin deficiency, exposure to aflatoxin B1 through dietary consumption, smoking, and the male gender (2). The 5-year cumulative risk for HCC in patients with cirrhosis ranges between 5 and 30%, depending on the cause (with the highest risk among those infected with HCV), region or ethnic group (17% in the United States and 30% in Japan), and stage of cirrhosis (with the highest risk among patients with decompensated disease) (6).

The stagnating prognosis of HCC is contributed in part by the limited treatment options. For early-stage HCC, curative treatment options involve resection and/or liver transplantation, with >50% 5-year overall survival. By contrast, for patients with late-stage unresectable disease or ineligible for transplantation, 5-year overall survival is <10%. Treatment options may include transarterial chemoembolization (TACE) and radiofrequency ablation. In terms of systemic treatment, the multi-kinase inhibitor sorafenib is the only first-line agent for HCC, but it only extends survival by 2.8 months in late-stage patients (7). Regorafenib (a multi-kinase inhibitor) (8) and nivolumab (a PD-1 inhibitor) (9) were recently approved as second-line treatment in patients who progressed on sorafenib, bringing the total number of approved systemic agents to three, still far fewer than most other cancer types.

Hepatocarcinogenesis is a complex, multi-step process of histologic transformation of normal hepatocytes to HCC, involving the accumulation of epi/genetic alterations in hepatocytes (10). In this review, we summarized some of the challenges in the diagnosis and clinical management of HCC, assessed the current status of circulating cell-free DNA (cfDNA) as a “liquid biopsy” in HCC, and discussed the outlook of how circulating cfDNA may address some of the challenges in the clinic.

## Clinical Challenges in Liver Cancer

In the context of precision medicine, one of the biggest challenges in HCC clinical management is the frequent lack of tumor tissue for molecular profiling. Tumor materials may be obtained during resection and/or transplantation. However, these procedures are restricted to patients with early-stage, limited HCC (11). Unlike most other solid tumor types, the diagnosis of HCC does not always require the histopathologic analysis of tumor tissue. This is particularly true for patients with a known background of cirrhosis. In cirrhotic patients, HCC is frequently diagnosed on radiology alone or with serum alpha-fetoprotein (AFP) levels (11). Consequently, tumor materials for genetic profiling are unavailable for many unresectable HCC patients. Indeed, molecular profiling studies of HCC tumor tissues are frequently biased toward resectable tumors (12–23). Moreover, biopsy and surgical intervention are invasive procedures, and thus longitudinal sampling for the purpose of disease monitoring is clinically not feasible nor warranted in nearly all cases. An alternative source of DNA for molecular profiling would be highly desirable.

Early detection from screening programs has been reported to confer a survival benefit in HCC patients (24, 25). Currently, the most widely used blood-based biomarker is serum AFP. The clinical utility of AFP for diagnosis, even in the screening setting of high-risk patients, is limited by its lack of sensitivity and specificity. In fact, a substantial proportion of HCC patients do not display elevated serum AFP levels, while patients with chronic liver disease may also show elevated levels (26). Indeed, AFP is not currently recommended as the sole diagnostic marker (11).

Like virtually all solid tumors, variable levels of intra-tumor genetic heterogeneity with branched and parallel evolutionary patterns have been detected in HCC patients (27, 28). Genetic diversity appears to show spatial organization, with intrahepatic metastases showing rapid diversification at the distant site (28). HCC may also metastasize to distant organs, most frequently to the lungs, the lymph nodes, bone, and the adrenal glands. To add to the complexity, recurrent or multifocal HCC may represent intrahepatic metastasis, in which the malignant cells are disseminated from a single primary tumor or may represent independent (multi-centric) tumors (29). Clinical distinction between these two entities is important as intrahepatic metastases are likely to be more poorly differentiated and aggressive. Molecular analysis based on whole-genome sequencing in a recent study of 20 patients with synchronous or metachronous disease within the liver found that 65% (13/20) patients had evidence of multi-centric tumors (29). The direct clinical implication of intra-tumor genetic heterogeneity and multi-centricity is that a single biopsy may be inadequate to give a representative portrait of the epi/genomic landscape of the disease.

## What is Circulating cfDNA?

**Figure 1:**
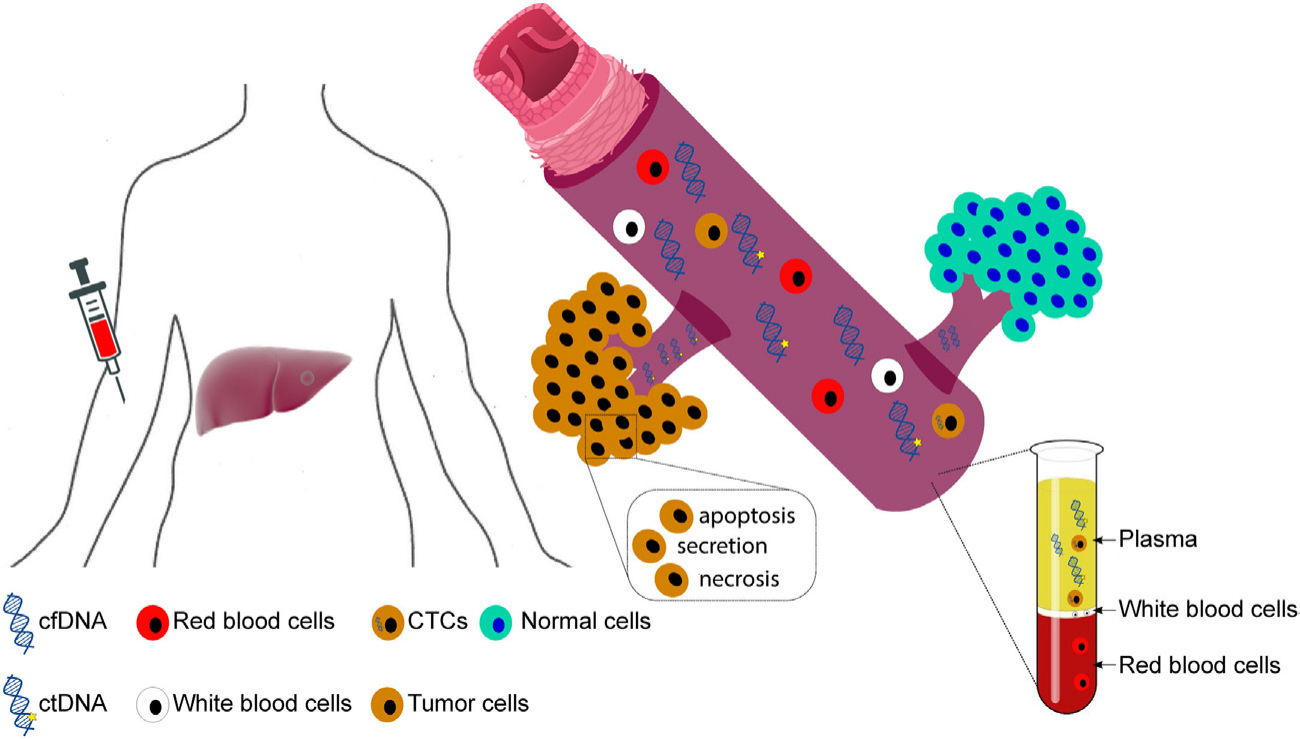
Circulating cell-free DNA (cfDNA) and circulating tumor cells (CTCs). Figure illustrates the origin of circulating cfDNA and CTCs. Circulating cfDNA may be released by apoptotic and necrotic cells, as well as through the secretion of living cells. In cancer patients, the fraction of tumor-derived cfDNA comprises circulating tumor DNA (ctDNA).

Under the general nomenclature of “liquid biopsy,” the two main types are circulating tumor cells (CTCs) and cfDNA (Figure 1). CTCs refer to the intact tumor cells released into circulation. Analysis of CTCs offers the opportunity to study their behavior in experimental systems and to study them on the DNA, RNA, and protein levels (30). However, current methods detect only a few CTCs in a single blood draw of 7.5 ml (31, 32), limiting their clinical utility.

By contrast, circulating tumor DNA (ctDNA), the fraction of cfDNA derived from tumor cells, appears to be more readily detectable than CTCs (33). Most circulating cfDNA fragments are 160–180 base pair fragments, roughly the size of a mononucleosomal unit, suggesting that they are largely released by apoptotic cells (34, 35). In most cases, white blood cells are the biggest contributor of cfDNA in the plasma (36). By contrast, ctDNA should in theory harbor the same epi/genetic alterations as the originating tumor cells. Interestingly, HCC patients have been reported to have aberrantly shorter and longer cfDNA fragments, with the shorter fragments likely to have derived from tumor cells, while the longer fragments are hypothesized to have derived from necrotic cells (35).

Studies of the potential clinical utility of cfDNA are based on the hypothesis that abnormal forms of circulating cfDNA are more likely to be present in cancer patients. Both plasma-derived and serum-derived cfDNA/ctDNA have been and are being investigated as potential blood-based biomarkers for diagnosis, prognosis, and as a surrogate for molecular profiling in HCC (Table 1).

**Table 1:** List of studies reporting on the analysis of circulating cfDNA in HCC patients.

Study	Method	cfDNA levels	Methylation of ctDNA	Genetic alterations of ctDNA	Reference
Wong et al.	Methylation-specific PCR of *p16*		√		(37)
Wong et al.	Methylation-specific PCR of *p15* and *p16*		√		(38)
Yeo et al.	Methylation-specific PCR of *RASSF1A*		√		(39)
Iizuka et al.	Real-time PCR of *GSTP1*	√			(40)
Ren et al.	cfDNA quantification and allelic imbalance of 8p	√			(41)
Wang et al.	Methylation-specific PCR of *GSTP1*		√		(42)
Tangkijvanich et al.	Combined bisulfite restriction PCR for methylation of LINE-1 repetitive sequences		√		(43)
Tokuhisa et al.	Real-time PCR of *GSTP1*	√			(44)
Zhang et al.	Methylation-specific PCR of *RASSF1A, p15* and *p16*		√		(45)
Chan et al.	Methylation-specific PCR of *RASSF1A*		√		(46)
El-Shazly et al.	Quantitative real-time PCR of Alu repeats	√			(47)
Chen et al.	Quantitative real-time PCR of beta-actin genomic DNA fragments	√			(48)
Huang et al.	Quantitative real-time PCR of cfDNA	√			(49)
Chan et al.	Whole-genome sequencing			√	(50)
Chan et al.	Genome-wide bisulfite sequencing		√	√	(51)
Bettegowda et al.	Safe-SeqS of tumor-specific somatic mutations			√	(33)
Jiang et al.	Whole-genome sequencing			√	(35)
Ono et al.	Real-time PCR of tumor-specific structural variations			√	(52)
Ono et al.	Whole-exome sequencing			√	(52)
Sun et al.	Genome-wide bisulfite sequencing		√	√	(36)
Xu et al.	Whole-genome sequencing			√	(53)
Wen et al.	MCTA-Seq		√		(54)
Huang et al.	ddPCR of four mutation hotspots			√	(55)
Liao et al.	Targeted sequencing of *TERT, CTNNB1*, and *TP53* mutation hotspots			√	(56)
Huang et al.	Whole-exome multi-region sequencing and targeted deep sequencing			√	(27)
Xu et al.	Molecular inversion probes for aberrant methylation		√		(57)
Ng et al.	Targeted sequencing of 46 genes frequently altered in HCC			√	(58)

*PCR, polymerase chain reaction; ddPCR, digital droplet PCR; cfDNA, cell-free DNA; Safe-SeqS, safe-sequencing system; MCTA-Seq: methylated CpG tandems amplification and sequencing; ctDNA, circulating tumor DNA*.

## Quantitative Analysis of cfDNA in Hepatocellular Carcinoma

The initial description of elevated cfDNA concentration in cancer patients (59, 60) led the scientific community to further investigate the clinical utility of cfDNA concentration. In HCC, one of the first studies evaluating serum cfDNA concentrations in 52 HCV-associated HCC patients found that HCC patients had increased cfDNA concentrations compared to HCV carriers without known HCC and HCV-negative non-HCC individuals (40) (Table 1). The authors confirmed their findings in a follow-up study with a larger cohort of 96 HCV-associated HCC patients and 100 HCV carriers without known HCC (44). Since then, studies have reported the concentration of cfDNA in serum or plasma to be 3–4 times higher in HCC patients compared to patients with chronic hepatitis (44, 49, 61) and nearly 20 times that of healthy controls (49). Several studies, including our own, have also reported that high cfDNA concentration was associated with larger tumors (40, 41, 58), higher tumor grade (40, 58), shorter overall survival (41, 44), and may serve as predictive biomarker for distant metastasis after curative surgery (44).

Attempts to use cfDNA quantification as a diagnostic tool have been more mixed, with one study reporting that cfDNA detection using quantitative PCR could discriminate HCC from normal controls with 90% sensitivity and 90% specificity (49). However, a meta-analysis found that cfDNA alone lacked robustness as the sole diagnostic tool for HCC, but its performance could be enhanced when used in conjunction with serum AFP levels (62). Importantly, quantitative analysis of cfDNA, as opposed to ctDNA, does not provide information into the biological characteristics and potential molecular targets of the HCC. Distinguishing ctDNA from cfDNA would require alternative approaches such as genomic and/or epigenomic analyses focusing on tumor-specific alterations.

## Methylation Analysis of ctDNA in Hepatocellular Carcinoma

DNA methylation is a common heritable mark of epigenetic regulation in eukaryotic organisms involving the covalent transfer of a methyl group to the C-5 position of the cytosine ring of DNA by DNA methyltransferases (63). Epigenetic mechanisms have been shown to play a pivotal role in the carcinogenesis of human tumors (64). It has been demonstrated that aberrant methylation of CpG islands serves as an important mechanism for the inactivation of tumor suppressor genes involved in hepatocarcinogenesis (42, 65–67).

The first studies to use methylation status as a tumor-specific marker to detect ctDNA in HCC patients investigated the methylation status of the *p15* and *p16* genes (37, 38) (Table 1), both of which are frequently abrogated in human neoplasms (68, 69). In these studies, the authors found that *p15* and *p16* were methylated in 16% (4/25) and 59% (13/22), respectively, of the plasma/serum DNA (37, 38). Importantly, the authors demonstrated that all cases with evidence of *p15/16* methylation in the plasma/serum DNA also showed evidence of methylation in the corresponding tumors (37, 38), suggesting that ctDNA reflects the epigenetic status of the originating tumors. On the other hand, not all HCC tumors with *p15/p16* methylation were associated with the equivalent methylation status in the plasma/serum DNA, underscoring the observation that ctDNA likely only accounts for a subset of cfDNA (36). Subsequent studies expanded the investigations to hypermethylation of the glutathione *S*-transferase P1 (*GSTP1*) and ras association domain family 1A (*RASSF1A*) genes (39, 42, 45, 46) and hypomethylation of LINE-1 repetitive sequence (43). While hypermethylation of *GSTP1* was detected in 50% (16/32) of the cfDNA (42), it appears that *RASSF1A* methylation was a more sensitive marker, with 70–93% of the sera of HCC patients showing evidence of hypermethylation (45, 46). It has also been reported that aberrant methylation in ctDNA may identify AFP-negative HCC (46). In agreement with the quantification of cfDNA, methylation studies have found that aberrant methylation is associated with increased risk of metastasis or recurrence (38), larger tumors (39, 43), and worse prognosis (43, 46).

More recent studies have taken advantage of NGS-based genome-wide survey of the methylation landscape to identify diagnostic and prognostic methylation markers suitable for ctDNA profiling. One such study described the use of methylated CpG tandems amplification and sequencing for the genome-wide detection of hypermethylated CpG islands in the cfDNA of HCC patients (54). The authors identified *TGS10, ST8SIA6, RUNX2*, and *VIM* as the best hypermethylated markers for the detection of small HCC (<3 cm) (54). Another study used The Cancer Genome Atlas methylation profiles of HCC tumors and an independent data set of normal blood leukocytes to construct a diagnostic prediction model using 10 methylation markers (57). When tested in the cfDNA, the diagnostic models created by these studies achieved 94% sensitivity and 89% specificity in distinguishing HCC patients from cirrhotic or normal controls (54) and >83% sensitivity and >90% specificity in distinguishing HCC patients from normal controls (57). Both studies detected aberrant methylation in a subset of or all AFP-negative HCC (54, 57). One of the studies also showed that the diagnostic model could also differentiate HCC patients from those with liver diseases such as HBV/HCV infection and fatty liver disease and that the model scores correlated with tumor burden, treatment response, and disease stage (57).

On a more global scale, genome-wide hypomethylation is frequently observed in human cancer (51, 70). Genome-wide bisulfite sequencing of plasma-derived cfDNA showed that plasma methylation profiles among healthy individuals are fairly stable and did not show evidence of global hypomethylation (51). By contrast, plasma-derived cfDNA from predominantly HBV-positive and Barcelona Clinic Liver Cancer (BCLC) stage A HCC patients showed that a median of 34.1% (IQR 2.5–56.7%) of the genome was hypomethylated (51). These results illustrate that global hypomethylation associated with HCC is reflected in the cfDNA (51). Furthermore, cfDNA global hypomethylation as a marker to distinguish HCC patients from healthy individuals was reported to be 81% sensitive and 93% specific (51). Deconvolution of the methylation profiles suggests that while the liver contributes to ~10% of cfDNA in healthy individuals, the fraction rises to 24% (IQR 19.0–44.0%) in HCC patients (36). The variability among HCC patients is consistent with the substantial inter-individual variability in the proportion of hypomethylated genome (51), as it would be expected that the sensitivity to detect hypomethylation would be reduced in HCC patients with low fraction of ctDNA.

Besides being investigated as a diagnostic aid, methylation levels may also serve as a disease monitoring tool. For instance, rising serum concentrations of methylated *RASSF1A* have been reported in HBV carriers progressing to HCC (46). On the other hand, while it appears that methylation markers in the cfDNA are linked to the tumor, it is unclear whether they are derived directly from HCC tissue (54).

## Genetic Analysis of ctDNA in Hepatocellular Carcinoma

Genomic characterization of HCC to the base pair resolution has revealed that no two HCCs harbor the same repertoire of somatic genetic alterations (13, 15, 16, 21–23). However, the somatic genetic alterations frequently found in HCC converge onto several main pathways, namely, p53 signaling (e.g., *TP53* and *CDKN2A)*, Wnt/β-catenin pathway (e.g., *CTNNB1* and *AXIN1*), chromatin remodeling (e.g., *ARID1A, ARID1B, ARID2, BAP1, MLL, MLL3*, and *PBRM1*), response to oxidative stress (e.g., *KEAP1* and *NFE2L2*), telomerase maintenance (e.g., *TERT* promoter mutation, HBV integration into *TERT, TERT* fusion genes, and *TERT* copy number amplification), among others (21, 23). With therapeutic decisions being increasingly genotype-based, a number of studies have investigated the detection of ctDNA on the basis of somatic genetic alterations in HCC.

The proportion of HCC patients with detectable ctDNA based on somatic genetic alterations varies wildly between studies. Using direct sequencing, the *TP53* R249S mutation could be detected in 26% of the plasma DNA in an African HCC patient cohort (71). Using NGS investigating hotspot mutations in *TERT* promoter, *CTNNB1*, and *TP53*, tumor-associated mutations were detected in 8/41 (20%) plasma samples in a predominantly HBV-positive cohort (56) (Table 1). Interrogating *TP53* c.747G>T (p.R249S), *CTNNB1* c.121A>G (p.T41A) and c.133T>C (p.S45A), and *TERT* c.-124C>T promoter mutations using digital droplet PCR (ddPCR), ctDNA could be detected in 27/48 (56%) of the predominantly HBV-positive and BCLC stage A patients (55). Furthermore, copy number analysis found that 13/31 (42%) of HCC patients had detectable copy number alterations in the plasma (53). In the seminal study by Bettegowda et al., it was reported that the fraction of advanced stage HCC patients with at least one detectable somatic mutation in the plasma was 75% (3/4), which was similar to other solid tumors of non-brain origin (33).

The variability in the proportion of HCC patients with detectable ctDNA appears to be related to cohort composition and geographical locations, as well as the methodologies for detection. Nonetheless, consistent with cfDNA quantification and methylation studies, detectable ctDNA on the basis of tumor-specific genetic alterations is associated with well-established clinicopathologic parameters such as tumor size, AFP, and vascular invasion (52, 53, 56), as well as recurrence and extrahepatic metastasis (52, 56). Our own experience is largely consistent with these observations. In a recently published study of 30 HCC patients, we demonstrated that somatic mutations in genes frequently genetically altered were detected in 27% (8/30) of patients and in 86% (6/7) of patients with large tumor (≥5 cm diameter) or metastatic disease (58). We also observed that detectable ctDNA on the basis of tumor-specific mutations was positively correlated with tumor size and Edmondson grade, although not with BCLC stage or AFP levels (58).

The fraction of ctDNA in cfDNA, estimated based on the fraction of sequences harboring tumor-associated mutations, also appears to vary substantially between patients. Among the patients with detectable ctDNA, ddPCR of four hotspot mutations in *TP53, CTNNB1*, and *TERT* promoter found that the mutant allele fraction ranged from 0.33 to 23.7% (55). Our own NGS-based study identified mutant allele fractions from 0.06 to 45% in the cfDNA among patients with detectable ctDNA (58). Based on copy number alterations and/or methylation, it was estimated that a median of 24.0% (range: 4.3–71.4%) of cfDNA was tumor-derived (36, 50). However, even between three HCC patients with advanced stage disease, there was enormous variability, with 7.2, 15, and 7,910 mutant fragments detected per 5 ml of plasma (33).

Analysis of tumor-specific genetic alterations has also allowed us to determine how well ctDNA would reflect the biology of the tumors. Indeed, whole-genome sequencing performed in the plasma-derived cfDNA suggests that the copy number profiles of plasma DNA highly resemble those of the matched primary tumor (35, 50). Furthermore, hotspot mutations detected in the plasma DNA were almost always associated with the detection of the same mutations in the corresponding tumors (56). In our own study, comparing plasma-derived cfDNA and synchronously collected frozen biopsies from the primary tumor, we found that 87% (80/92) of the mutations were captured in the cfDNA among the seven patients in whom the largest tumor was ≥5 cm or was associated with metastasis (58). Importantly, we found that the proportion of mutations captured in the matched primary tumors of these patients was similar (95%, 87/92). Our results suggest that ctDNA reflects the biology of the primary tumors in patients with high disease burden. These findings are particularly important in the context of HCC, given that patients with high disease are the least likely to undergo surgical resection.

The more exciting recent development is the potential for ctDNA to circumvent the intra-tumor genetic heterogeneity in HCC (27, 28). Its potential was demonstrated with the use of whole-genome sequencing of the cfDNA from four HCC patients before surgery. The analysis revealed that 15–94% tumor-associated single nucleotide variants could be detected in the cfDNA, even at the very limited depth of 17× (50). Similarly, whole-exome sequencing of a patient with combined HCC/cholangiocarcinoma found that 63% of the tumor-associated mutations were observed in the serum DNA (52). However, the tumor and the serum samples for this patient were collected 2 years apart, which makes it difficult to assess whether the differences reflect the clonal evolution that may have occurred through two rounds of TACE (52). It may be argued that a synchronously collected cfDNA sample would have been more representative of the tumor. Indeed, synchronously collected cfDNA samples were representative of the primary tumors, but only in patients with high disease burden (58). We also found instances where mutations with low variant allele fraction (therefore almost certainly subclonal) in the primary tumors were readily detectable only in the cfDNA (58), suggesting that there is potential for ctDNA to circumvent the intra-tumor genetic heterogeneity.

The most comprehensive study to date to address the question whether cfDNA captures intra-tumor heterogeneity was conducted by multi-region whole-exome sequencing of five patients (27). The authors found that the cfDNA captured most of the mutations that were heterogeneous between tumor regions, but only given the knowledge of the mutations present in the tumor (27). On the other hand, agnostic to the mutations in the tumor, the authors concluded that cfDNA was insufficient in capturing the heterogeneity of the disease but could be used to identify actionable mutations that were ubiquitous in the tumor (27).

Given the apparent correlation between ctDNA fraction and disease burden (33, 58), genomic profiling is also being investigated as a disease monitoring tool and for the detection of recurrence prior to clinical recurrence. Similar to cfDNA levels and methylation analysis of ctDNA, quantification of ctDNA on the basis of somatic genetic alterations showed that the ctDNA level mirrors response to treatment and disease progression, in that the ctDNA level dropped or completely disappeared after resection and rose prior to recurrence or metastasis (50–52). By contrast, in patients that remained in long-term remission, ctDNA was not detectable in long-term follow-up cfDNA samples (51, 52).

## Outlook: Addressing Clinical Problems in HCC

**Figure 2:**
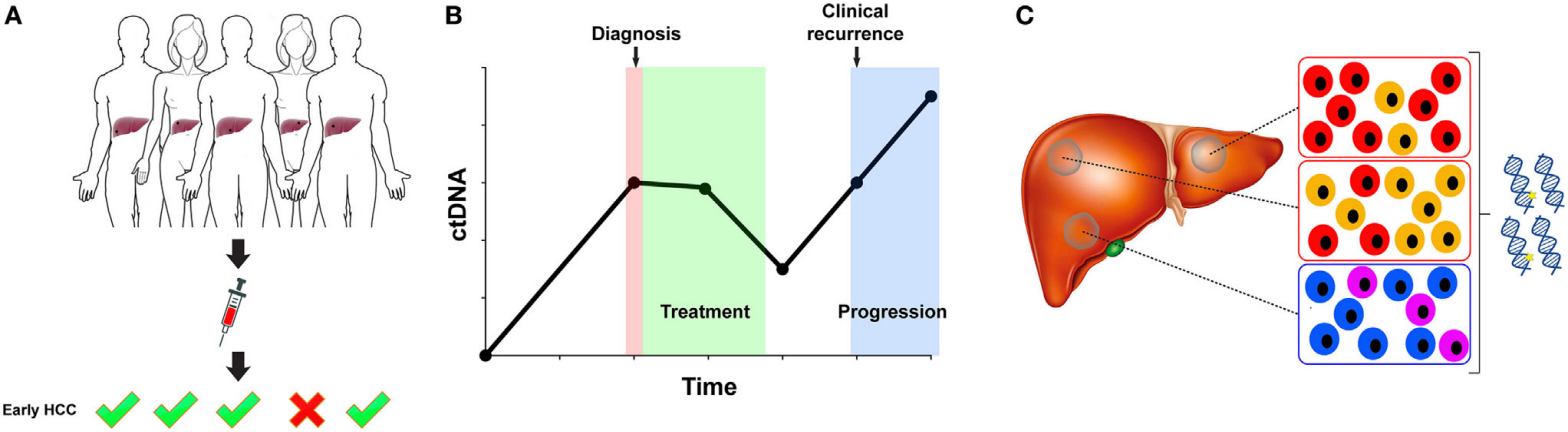
Circulating tumor DNA (ctDNA) for early detection, disease monitoring, and molecular profiling. **(A)** ctDNA is being investigated as a marker for the detection of early hepatocellular carcinoma (HCC). A suitable marker for early detection in the screening setting would require excellent specificity. **(B)** The fluctuation of ctDNA level may be informative in assessing response to treatment and in detecting minimal residual disease. **(C)** ctDNA profiling may circumvent sampling biases resulting from intra-tumor genetic heterogeneity and multi-centric HCC.

The characterization of ctDNA in HCC patients has focused on three main aspects: its potential for risk prediction or early cancer detection in a screening setting, for response to treatment and recurrence monitoring, and as a surrogate for tumor molecular profiling (Figure 2). With serum AFP levels being the only widely used but insensitive blood-based biomarker for HCC, a more sensitive and specific biomarker is highly desirable. While cfDNA level correlates with the disease stage and is easy to measure, it alone also lacks sensitivity and specificity (62). Moreover, elevated cfDNA level is not unique to HCC, as cfDNA level is also elevated in individuals with diabetes, stroke, acute trauma, pregnancy, among others (72).

Despite the increased sensitivity in detection methods, establishing the utility of ctDNA for risk prediction or early cancer detection in a screening setting remains challenging. Overcoming the challenges posed by the low fraction of ctDNA within cfDNA in early-stage HCC would be crucial. Thus far, a study of 50 HCC patients with repeated serum DNA sampling suggested that aberrant methylation could be detected up to 9 years prior to the diagnosis of HCC (45). There are a number of other anecdotal examples to suggest that the detection of epi/genetic alterations in ctDNA may predate clinical diagnosis. For instance, whole-genome sequencing of two cases without known HCC revealed the presence of copy number alterations in the plasma cfDNA, and the patients were diagnosed with HCC 3–4 months later (35). Large case–control studies required to establish the utility of ctDNA in early cancer detection are inherently difficult to carry out, as they will have to be prospective, will involve repeated sampling at regular intervals, and with long-term follow-up of the patients.

Developing a ctDNA test for early cancer detection would involve the selection of an appropriate marker broadly applicable for a given cohort. Highly recurrent hotspot mutations such as *TP53* R249S, *CTNNB1* amino acids D32, S33, S37, T41, and S45, and *TERT* c.-124C>T promoter mutations (13, 15, 16, 21–23) may serve as potential markers for HCC detection. However, the discovery of frequent *TERT* promoter mutation in cirrhotic pre-neoplastic lesions (73) suggests that it may lack specificity in a screening setting. An intriguing possibility could be to detect ctDNA on the basis of shorter fragment sizes (35), although it is currently unclear whether shorter fragment size is a universal feature of ctDNA.

Compared to early detection, ctDNA for response and recurrence monitoring is closer to clinical application. In other cancer types, ctDNA is being used to detect minimal residual disease (74) and to track the emergence of drug resistant clones (75). HCC patients often undergo repeated rounds of therapeutic interventions such as TACE and ablation, with recurrence occurring several years later. Clonal evolution of the disease means that the genetic information obtained from tumor tissue at diagnosis or resection may become outdated by the time of recurrence. ctDNA profiling makes longitudinal monitoring of clonal evolution possible and may assist in the detection of micrometastatic disease (30). However, it should be noted that the timing of sample collection is an important consideration in the interpretation of the analysis, as it has been shown that serum ctDNA peaks 4 weeks after TACE (52).

Circulating tumor DNA profiling holds promise as a surrogate to overcome the challenges posed by intra-tumor genetic heterogeneity. It has been hypothesized that the detection of *TERT* promoter, *CTNNB1*, and *TP53* mutations in the plasma cfDNA but not in the HCC tumor may be attributed to intra-tumor genetic heterogeneity (55, 56). However, similar to the challenges faced in early detection, the ability to capture all somatic genetic alterations is hampered by the sensitivity of current technologies. For instance, whole-exome sequencing of a single HCC patient suggests that 63% of the mutations were shared between the resected tumor and the serum cfDNA 2 years post-surgery, but only 27% of the mutations would have been detected had the samples been analyzed independently (52). Similar observations were made in a multi-region whole-exome sequencing study of five HCC patients (27), highlighting the technical challenges in using ctDNA as a surrogate for molecular profiling in the absence of tumor tissue. However, it should be noted that the patients included in the studies had resectable disease. Given the correlation of ctDNA and tumor burden, it is conceivable that a larger fraction of alterations would be represented and detectable in the ctDNA in late-stage patients. Indeed, we found that cfDNA profiling nearly recapitulated the repertoire of mutations detected in the primary tumors of patients with high disease burden (58), suggesting that the use of cfDNA for genetic profiling when biopsy is unavailable may be feasible in this subset of patients.

Currently, there are no widely accepted predictive markers for response to sorafenib or regorafenib. Response to immunotherapy such as nivolumab has been associated with tumor mutational burden (76). In fact, microsatellite instability status has recently been approved as an indication for nivolumab treatment of metastatic colorectal cancer (76). A subset of HCC exhibits a hypermutator phenotype (12, 21). With nivolumab approved as a second-line treatment, assessing mutational burden in the ctDNA of patients with advanced HCC should be feasible and may provide a predictive marker for response. Among our cohort of 30 patients, one HCC biopsy displayed a hypermutator phenotype, and the phenotype was clearly observed in the cfDNA (58). In addition, molecular analyses of ctDNA may uncover ubiquitous (i.e., present in all cancer cells) targetable genetic alterations that may quality patients for experimental therapy or off-label use. In a cohort of 70 HCC patients, 39% (27/70) harbored potential targetable alterations, including *TSC1/TSC2* mutations (mTOR inhibitors), *EGFR* mutations (gefitinib and erlotinib), *CCND1* amplifications and *CDKN2A* deletions or mutations (palbociclib), *ATM* mutation (olaparib), and *MET* amplification (tivantinib) (27). Using a more restricted targeted sequencing consisting of 46 coding and non-coding genes frequently altered in HCCs, we also detected a *TSC2* frameshift mutation in the cfDNA and primary tumor of a metastatic patient (58).

Thus far, cfDNA profiling of HCC has focused on blood-based analysis (plasma and serum). It is plausible that ctDNA may also be detected in other body fluids, such as urine. Indeed, a recent study reported the use of qPCR-based analysis of *TP53* c.747G>T (p.R249S) and aberrant methylation of the *GSTP1* and *RASSF1A* genes in the urine of 10 HCC patients to monitor recurrence during follow-up (77). Of the five patients who were confirmed by MRI for recurrence, all had detectable changes in the urine cfDNA. Similar to plasma-derived cfDNA, the authors reported up to 9 months of lead-time prior to radiological recurrence (77). Compared to blood, urine is even more readily available. Further investigations into the use of urine would certainly be of clinical interest.

Through the analysis of cfDNA quantity, methylation, and genomic studies of ctDNA, it is clear that ctDNA holds potential to address a number of outstanding questions in the clinical management of HCC. Studies carried out thus far have laid the foundation for future studies into the use of ctDNA in early detection, response assessment, and detection of minimal residual disease. Exploiting ctDNA profiling to overcome the challenges posed by intra-tumor genetic heterogeneity may also provide biological insights into hepatocarcinogenesis.

## Author Contributions

CN and SP conceived the study. CN, GDC, LT, and SP performed literature screening. CN and SP wrote the manuscript. All the authors reviewed and approved the final version of the manuscript.

## Conflict of Interest Statement

The authors declare that the research was conducted in the absence of any commercial or financial relationships that could be construed as a potential conflict of interest.
